# An experimental paradigm for studying EEG correlates of olfactory discrimination

**DOI:** 10.3389/fnhum.2023.1117801

**Published:** 2023-05-25

**Authors:** Ivan Ninenko, Daria F. Kleeva, Nikita Bukreev, Mikhail A. Lebedev

**Affiliations:** ^1^Institute for Cognitive Neuroscience, HSE University, Moscow, Russia; ^2^V. Zelman Center for Neurobiology and Brain Restoration, Skolkovo Institute of Science and Technology, Moscow, Russia; ^3^SensoryLab, Inc., Moscow, Russia; ^4^Faculty of Mechanics and Mathematics, Lomonosov Moscow State University, Moscow, Russia; ^5^Sechenov Institute of Evolutionary Physiology and Biochemistry of the Russian Academy of Sciences, Saint Petersburg, Russia

**Keywords:** olfaction, EEG, BCI, instructed-delay, olfactory processing

## Abstract

Electroencephalography (EEG) correlates of olfaction are of fundamental and practical interest for many reasons. In the field of neural technologies, olfactory-based brain-computer interfaces (BCIs) represent an approach that could be useful for neurorehabilitation of anosmia, dysosmia and hyposmia. While the idea of a BCI that decodes neural responses to different odors and/or enables odor-based neurofeedback is appealing, the results of previous EEG investigations into the olfactory domain are rather inconsistent, particularly when non-primary processing of olfactory signals is concerned. Here we developed an experimental paradigm where EEG recordings are conducted while a participant executes an olfaction-based instructed-delay task. We utilized an olfactory display and a sensor of respiration to deliver odors in a strictly controlled fashion. We showed that with this approach spatial and spectral EEG properties could be analyzed to assess neural processing of olfactory stimuli and their conversion into a motor response. We conclude that EEG recordings are suitable for detecting active processing of odors. As such they could be integrated in a BCI that strives to rehabilitate olfactory disabilities or uses odors for hedonistic purposes.

## 1. Introduction

Electroencephalography (EEG) is a well-developed method for non-invasive research of cortical processing. While the EEG approach has been successfully used in the studies of motor control, visual and auditory processing, the results of EEG investigations into the olfactory domain are rather mixed. In a pioneering study, Moncrief (1962) reported that a wide range of odors (essential oils, synthetic oils, and unpleasant chemicals) produced a general suppression in alpha activity, except for ylang-ylang that did not produce any effect. This observation was not fully supported by further studies, with some researchers reporting an increase in alpha activity (Van Toller et al., 1993) and others reported no significant change. Increases in theta activity were also reported (Klemm et al., 1992). Martin (1998) noted that the literature on EEG correlates of olfactory processing reports a diversity methods and findings, and the same assortment remains nowadays (Schriever et al., 2017; Gudziol and Guntinas-Lichius, 2019; Arpaia et al., 2022). Inconsistencies in the application of EEG methodology to olfaction impede the development of clinical markers for diagnosing and monitoring olfactory disorders and of olfactory-based BCIs that potentially could be useful to treat olfactory disabilities, such as anosmia, dysosmia and hyposmia.

The prospective BCI for olfaction should contain the following major components: an olfactory display for delivery of odors that evoke stable perceptions, an appropriately designed odor discrimination task, recordings of brain activity (e.g., EEG recordings in the method proposed here), and the approach for decoding olfactory information from neural activity.

Many of the previous studies relied on manual delivery of odors to participants. In this approach, odorants were usually stored in unlabeled bottles or on scented paper and then manually brought to the participant’s nose by the experimenter. Such manual delivery of odorants has many limitations, such as experimenter bias or uncontrolled amount of odorant (Lorig, 2000). Additionally, hand movement in direct proximity of the subject’s face can affect participants’ perception and add additional confounds to the experimental paradigm. For precise delivery of odors, a special tool called olfactometer is used. An olfactometer consists of a tube and/or a facial mask that releases odorants close to the nose. As far as natural odor delivery of odors and BCI applications are concerned, systems appear more appropriate that at are currently used in virtual-reality systems (Washburn et al., 2003; Richard et al., 2006; Munyan et al., 2016) and movie theaters (Kim et al., 2006; McGinley and McGinley, 2017) where they improve the sense of presence and the overall experience. Given this trend for using near-natural environments that incorporate multiple sensory modalities, including olfaction, we suggest that such environments could be complemented with measurements of brain activity and potentially with BCIs that enable direct communication between the environment and the brain, with practical applications such as relaxation (Serrano et al., 2016; Amores et al., 2018) and therapy (Aiken and Berry, 2015; Herz, 2021).

With respect to neural events related to olfactory processing that could be utilized for BCI purposes, basing decoding on EEG rhythms is one option, and the other is using event-related potentials (ERPs), such as P200 and N200 evoked by olfactory stimuli. Thus, the P200 component is a positive-going ERP that typically occurs around 200–300 ms after the onset of a stimulus (Sur and Sinha, 2009). The P200 is thought to reflect allocation of attention to the stimulus and processing of its relevance. Such responses may be evoked by odor stimuli and have become a topic of significant interest in recent studies (Arpaia et al., 2022). Additionally, in the studies of multisensory integration, olfactory stimuli modulate P200 components of the ERPs related to visual stimuli (Leleu et al., 2015). Several groups explored chemosensory event-related potentials (CSERPs) and olfactory event-related potentials (OERPs) in detail (Invitto and Mazzatenta, 2019). Thus, it was found that processing speed decreased with age for the P200 OERP component (Murphy et al., 2000). Additionally, OERPs deteriorate in patients with olfactory dysfunctions, which suggests their use to improve diagnostics of such disorders.

Regarding decoding algorithms suitable for olfactory-based BCIs, several research groups reported using neural networks to classify odors based on EEG data (Saha et al., 2013; Aydemir, 2017; Abbasi et al., 2019; Ezzatdoost et al., 2020). High decoding accuracy (from 87.5 to 94.1%) was achieved by applying continuous wavelet transform to EEG gamma band followed by a K-nearest neighbors classifier (Aydemir, 2017). Additionally, excellent accuracy (up to 97%) was achieved by the application of Hopfield neural network to multidimensional wavelet and power spectrum density features (Saha et al., 2013).

Olfactory task design is important to evoke EEG modulations that could be decoded by a BCI. In many previous studies, subjects did not perform any task apart from passively perceiving odors while their brainwaves were recorded. An active discrimination “Sniffin’ sticks” test could be performed, but only prior to OERP recordings during passive perception (Rombaux et al., 2007).

Based on these considerations, here we developed an experimental paradigm for olfactory perception in human participants, which paves way toward olfactory-based BCIs. In this methodology, a participant is comfortably seated in a home theater-like room while odors are delivered using an odor display mounted to the ceiling. With this automated odor delivery system, near-natural olfactory experiences can be created and combined with sensory stimuli of different modalities. In the behavioral paradigm that we tested, olfactory stimuli were incorporated in an instructed-delay task and electrophysiological recordings were conducted. We followed the methodological recommendations for EEG studies of olfaction (Lorig, 2000). EEG and respiration data were collected in 17 healthy participants. Our analysis of spatio-spectral EEG properties showed that neural patterns can be assessed that are exhibited during this instructed-delay odor-discrimination task.

## 2. A materials and equipment

### 2.1. Controlled delivery of odors

**FIGURE 1:**
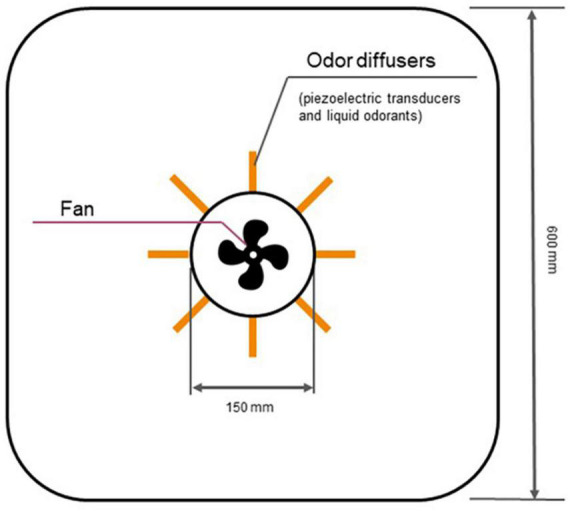
Schematics of olfactory display operation. The airflow is driven by a fan whereas liquid odorants are released under the control of piezoelectric transducers.

**FIGURE 2:**
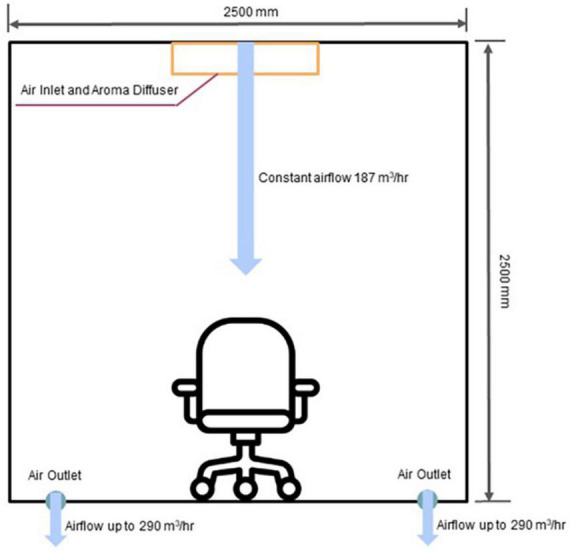
Schematics of the odor delivery and airflow in the room. Odors were delivered from the ceiling, and the exit points for the airflow were located on the floor. The participant’s chair was placed in the middle of the room.

**FIGURE 3:**
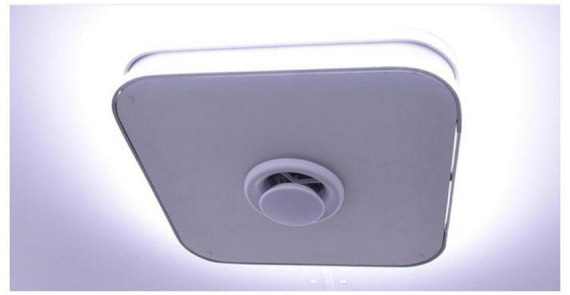
The olfactory display mounted to the ceiling.

For controlled and near-natural presentation of olfactory stimuli, we utilized an automated odor delivery system developed by Sensorylab Inc. The system uses piezoelectric transducers to release liquid odorants into the stream of air (Figure 1). The device controls the amount of odorant by regulating stimulus onset and offset. In this system, an odorant reached the subject approximately 400 ms after stimulus onset. The automatic odor delivery system was mounted on the ceiling in a room designed to serve as a home theater (Figure 2). The room was a cubic enclosure (2.5 × 2.5 × 2.5 m). A constant airstream descended from the ceiling, and odors could be added to this airstream under computer control. The airflow for the olfactory display was 187 m^3^/hr. The system’s air circulation capacity was 580 m^3^/hr. A participant was seated in a comfortable armchair in the middle of the room. A screen was mounted in front of the participant for the delivery of visual stimuli and entertainment, and the projector was mounted behind the participant. A photograph of the odor delivery system mounted on the ceiling is shown in Figure 3. A video showing odor delivery process is available in the Supplementary material.

### 2.2. Olfactory stimuli

The odorants were dissolved to achieve viscosity necessary for stable evaporation with piezoelectric transducers. Ethanol-based solvent was applied to natural coffee, perfume vanilla and citrus essential oil. Four liquid stimuli were used: vanilla, coffee, citrus, and odorless water (control stimuli). These flavors were selected in a pilot study where we sought for odors that were familiar to the general public. Besides the odors that were finally selected, we considered using rose water and mint, but chose to exclude these stimuli because Russian participants were usually unfamiliar with rose water and they associated mint with taste rather than smell.

### 2.3. EEG and respiration registration

Electroencephalography data were collected with a Smart BCI system (Mitsar, Russia) at 250 Hz sampling rate with impedance below 10 kΩ. Similar to the work of Martin (1998), 19 electrodes were positioned on the scalp according to the International 10–20 system with A1 + A2 ears reference. Respiration data were collected with TRSens temperature sensor for nasal-oral breathing and KARDi2-NP polygraph amplifier (Medical computer system, Russia). Data sampling was synced with the event markers for the button presses.

### 2.4. Joystick and button setup

**FIGURE 4:**
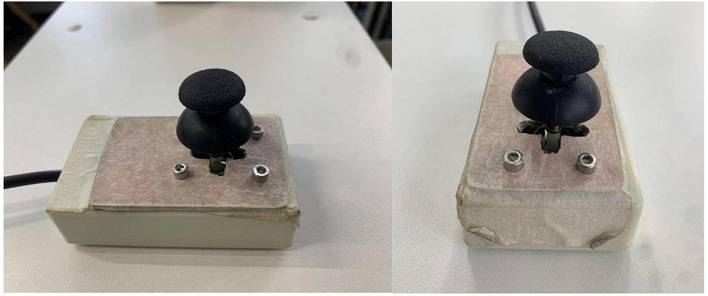
The two-dimensional joystick used for reporting the perceived smells. The joystick was a modified Arduino joystick which was mounted in a frame that limited movements to four possible report directions.

Participants operated a two-dimensional joystick to report the perceived odors. We adjusted an Arduino joystick for this purpose. The joystick was placed in a box that limited its movements to two dimensions (Figure 4).

**FIGURE 5:**
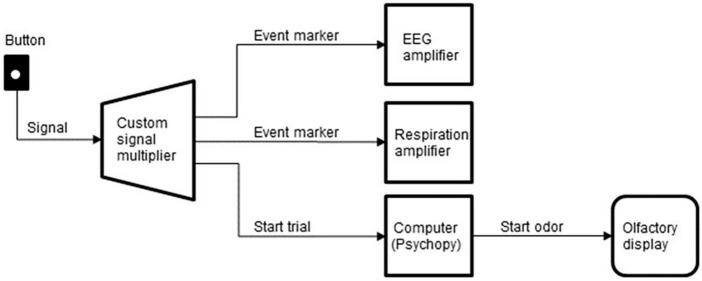
Schematics of the signal flow from the button to the other devices.

The subjects initiated each trial by pressing a button with the left hand, which started the sequence of task events and synced the system components. A custom signal multiplier distributed the signals from the button to three devices: the computer that ran the experimental sequence and controlled the olfactory display, the EEG amplifier, and the respiration amplifier (Figure 5).

## 3. Methods

### 3.1. Subjects

All subjects signed the informed consent forms prior to the experiment. The experiments were performed under the ethical approval from the Higher School of Economics Institutional Review Board (decision from 15th of February 2021). The participants did not receive any financial reward for this experiment.

We collected data in 17 healthy participants (age 22–44, median age = 31, females = 7). One subject’s EEG and respiratory data were excluded because the system malfunctioned during the recordings. All subjects filled the forms before and after the experiment. The first form included general sociodemographic information and some specific questions regarding olfaction. Four participants stated that they have an olfaction-related hobby (perfumery, winery etc.). None were professionally employed in fields that require special olfactory skills. None of the participants reported anosmia. One participant reported a broken nasal bone, which did not alter her odor perception.

### 3.2. Experiment task

**FIGURE 6:**
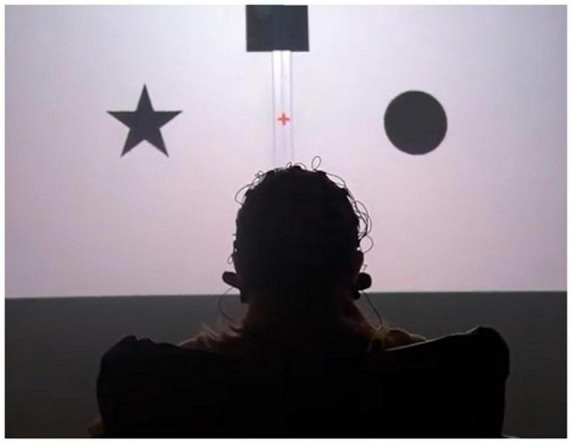
A photograph showing the participant sitting in front of the screen. The subject is viewing the stimuli associated with particular odors. The triangle is located at the bottom of the screen but is not visible in this picture.

The task was an instructed-delay task that required subjects to smell an odor and then transform this perception into a pointing movement performed with a joystick. By the experiment design, each odor (including no-smell condition) was associated with a visual label: a square, circle, triangle, or a star (Figure 6). Accordingly, the subjects reported their smell assessments by pointing to the appropriate label. Odor-label pairs were randomly generated for each participant and remained constant during each experimental session.

**FIGURE 7:**
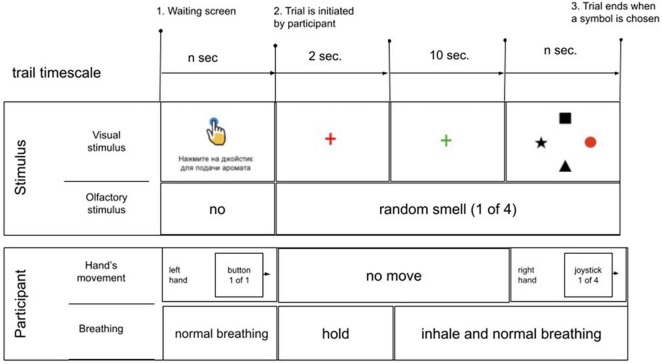
Schematics of the experimental sequence. In the beginning of each trial, an instruction (written in Russian) was shown on the screen: “press the joystick for aroma delivery”. Next, the subject pressed the button to start the sequence of task events. A red cross appeared on the screen for 2 s; the subject visually fixated the cross and held their breath. An odorant was delivered during this period. The cross then changed color into green, which instructed the subject to start inhaling. The subject continued breathing with a comfortable pace for 10 s. Finally, a display with four items was shown on the screen, and the subject pointed with the joystick to the item that corresponded to the perceived odor. If the session was a training session, the correct item was highlighted with red color. During the discrimination that followed all items were colored black.

Following the training (40 trials, 10× odor, random order), an odor discrimination session was run (80 trials, 20× odor, random order). After starting a trial, participants were instructed to hold their breath until the fixation cross turned green. Next, they pressed a button. Odor delivery started immediately after the button was pressed. Following a 2-s delay, the fixation cross changed color from red to green and the participant made the first inhale. After the subject assessed the odor for the subsequent 10 s, four objects appeared on the screen (at 0, 90, 180, and 270° positions); one of them represented the correct response. With this design, EEG and respiratory data were collected throughout all four task epochs: (1) no odor, (2) odor discrimination without any motor preparation, (3) motor preparation, and (4) peri-movement interval. After the entire experimental session was completed, participants verbally described their impressions of the odors delivered and other aspects of the task (Figure 7).

Participants were instructed to avoid unnecessary movements during the trial and use time before the next trial if there was a need to flex the neck or hands or conduct any other movement. These settings minimized the artifacts in the EEG recordings. The duration of the experiment varied across participants because they were allowed to make short breaks between the sessions.

### 3.3. Respiratory data preprocessing

All participants were instructed to start inhaling when the fixation cross turned green. An algorithm was developed to detect the exact moment the subject started to inhale. The algorithm utilized a sliding window (1 s width), and the inhalation start was determined as the curve deviation from a stationary value. Some participants failed to hold their breath on some trials and/or the inhalation onset was not sufficiently abrupt to be detected by the algorithm. Thus, in an individual with a broken nasal bone, only 58 inhalation onsets could be detected out of 120 algorithmically or by visual inspection. The oldest female participant (39 years) had a breathing pattern where we could not detect many of the inhalation onsets. The algorithm was adjusted to avoid false-positive detections. The number and percentage of detected inhales for each subject are provided in the Supplementary material. The median number of detected inhales was 106.5 out of 120 total trials.

### 3.4. EEG preprocessing

A low-pass filter with a 45-Hz cutoff, and a high-pass filter with a 2-Hz were applied to the EEG recordings. Independent component analysis (ICA) was performed to remove ocular artifacts. We used FastICA algorithm (Hyvärinen and Oja, 2000) where components were selected based on a high mutual information coefficient for Fp1 and Fp2 channels and an absence of the alpha-band peak.

## 4. Results

### 4.1. Behavioral results

Each subject performed 120 trials, with 40 training trials and 80 discrimination trials. The majority of participants (12 out of 17) successfully reported the presented odor in more than 90% of the trials. The median accuracy was 93.8% with standard deviation of 14.5%. The weakest result was 35 correct reports out of 80 trials (43.8%) in a male participant (age = 40). His performance accuracy was still significantly above the chance level of 25%. Performance accuracy across the participants is provided in the Supplementary material.

**FIGURE 8:**
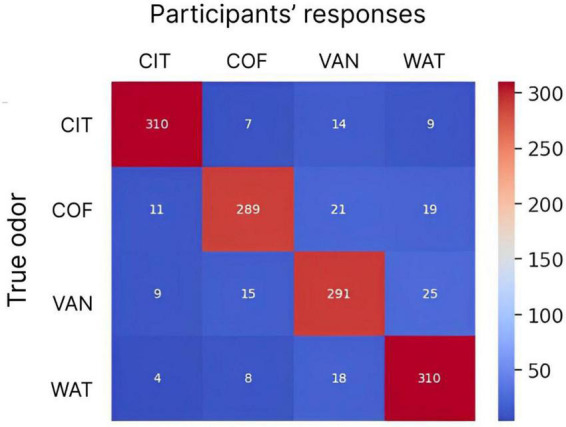
The confusion matrix for participants’ responses and actual odors being delivered. Numbers indicate the counts of responses for different delivered odors. Each odor was delivered 340 times (20 trials per odor for 17 subjects). The color scale is from blue (minimum) to red (maximum). CIT, COF, VAN, and WAT are citrus, coffee, vanilla and water, respectively.

To assess the discrimination performance for different odors, a confusion matrix was calculated, where the matrix rows represented odors being presented, the columns represented odors being reported, and the values were report counts (Figure 8). In this analysis, each subject performed 20 discrimination trials for each odor, so there was a total of 340 trials per odor for all 17 subjects. The confusion matrix showed an overall good performance for all subjects. The mean success rate was 93.8% with standard deviation of 14.5%. The best discrimination performance was for citrus odor and odorless water (310 correct responses out of 340 trials) and errors occurred most often for coffee (51 errors out of 340 trials).

**FIGURE 9:**
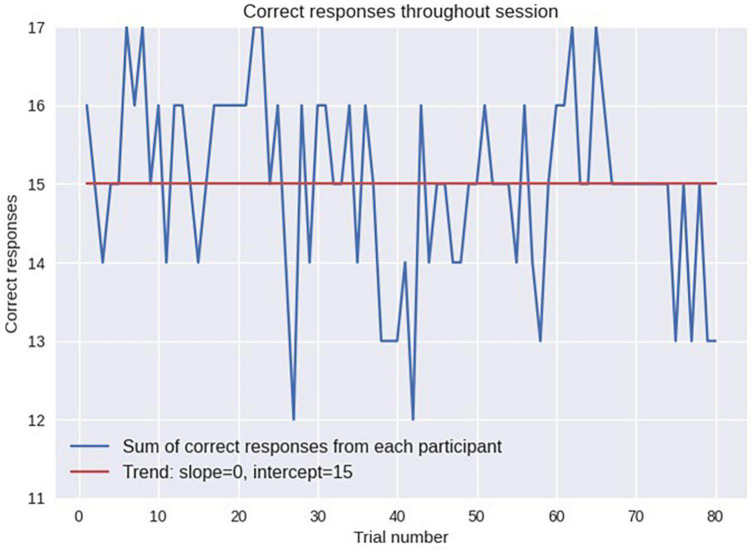
Performance accuracy evaluated as the number of subjects who responded correctly on each trial. No significant trend (from the first trial to the last) was found.

**FIGURE 10:**
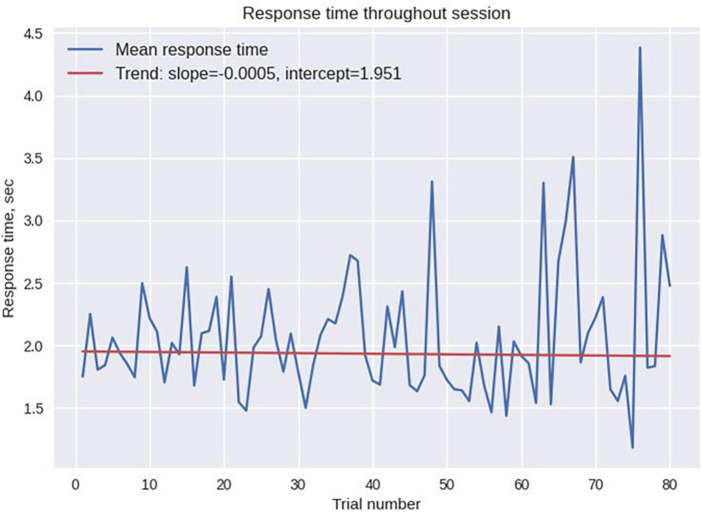
Mean response time across all participants for each trial. No significant time trend was found.

The statistics of odor discrimination was assessed with a chi-squared test applied to the confusion matrix (Table 1). Additionally, pre-whitened Mann–Kendall test for all the correct responses from each subject per each trial (Figure 9) revealed the absence of a significant trend (*z* = −1.5609, *p* = 0.1186), as well as the test of the mean response time (*z* = −0.3302, *p* = 0.7412, Figure 10). The statistical testing of individual response times demonstrated significant decrease in response times for some of the participants: 1R912 (*z* = −3.9793, *p* < 0.001), 5V005 (*z* = −3.1157, *p* < 0.05), 9V6NM (*z* = −3.6661, *p* < 0.001), FK7CK (*z* = −4.7583, *p* < 0.001). The only significant increase was found for participant 7PF8Z (*z* = 2.1251, *p* < 0.05).

**TABLE 1:** The results of the chi-squared test for the confusion matrix demonstrating the independent perception of different odors.

Stimulus	Power divergence	*p*-value
Citrus	819.509	<0.001
Coffee	732.524	<0.001
Vanilla	651.837	<0.001
Water	707.711	<0.001

Following the discrimination session, subjects were asked to fill in the form and provide names for the odors that were delivered. All participants correctly named odorless water and the majority of them (10 out of 17) named coffee. Yet only 5 and 3 subjects could correctly name citrus and vanilla odors, respectively.

### 4.2. Inhalation-related components

**FIGURE 11:**
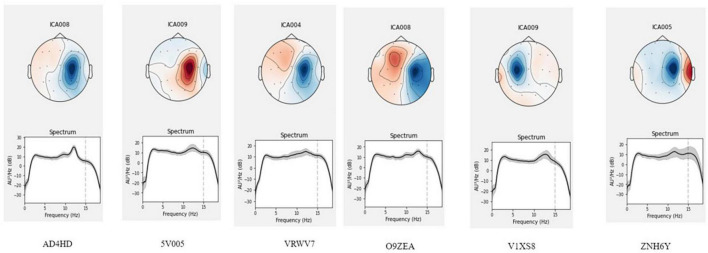
Location of the olfactory related component for six participants. The 7-s epochs after the inhale onset were analyzed, which preceded motor activity. ICA with the low-path filtering below 20 Hz allowed to extract an olfactory related component localized around the C4 lead (C3 for one participant).

A comprehensive analysis of EEG patterns in all participants is beyond the scope of this methodological article. We present several examples of analysis instead. In the analysis of EEG patterns representing odors, we focused on determining an independent inhalation-related component. We examined the 7-s epochs following the inhalation onset (within a 10-s period of odor processing and prior to symbols being displayed on the screen, thus prior to any motor activity). The initial attempts of ICA performed on bandpass-filtered signal between 2 and 40 Hz resulted in obtaining a great number of components characterized by high-frequency activity that were not time-locked to the inhalation onset. Therefore, we chose to analyze only the low-frequency EEG bands. When an ICA was applied to the low-pass (below 20 Hz) filtered EEG, we found components that were time-locked to the inhalation in six subjects. These components were localized around the C4 electrode (C3 for one subject), and their spectra had peaks at the alpha frequency (10–12 Hz). Figure 11 demonstrates localization and spectrum analysis of these components.

**FIGURE 12:**
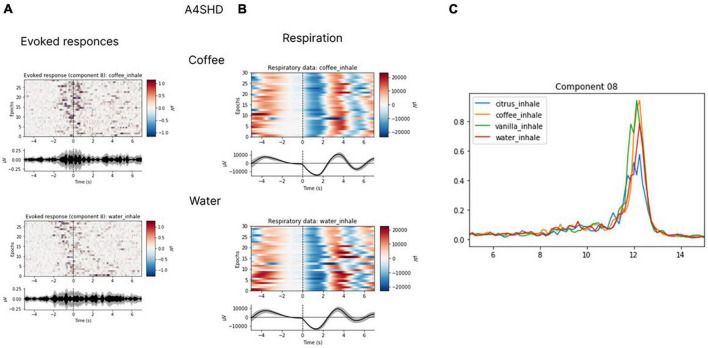
Evoked responses **(A)**, respiratory data **(B)** and spectral properties **(C)**. Evoked responses and respiratory data for coffee and water for participant A4SHD. The analysis was conducted for the 7-s epochs after the inhale onset, prior to hand movements. ICA with the low-path filtering below 20 Hz (*U* = 3200, *p* < 0.008 for a peak around 10 Hz in “coffee” and “water” conditions is significant at Bonferroni collected level).

**FIGURE 13:**
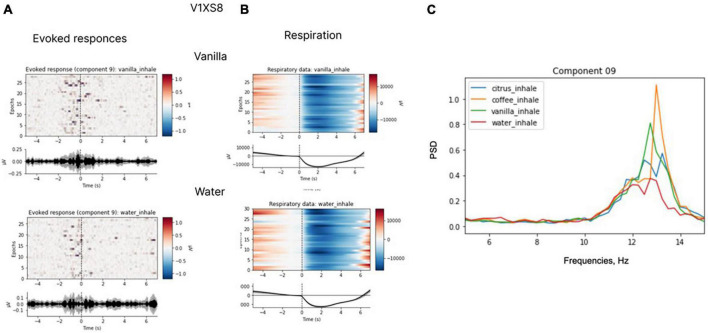
Evoked responses **(A)**, respiratory data **(B)** and spectral properties **(C)**. Evoked responses and respiratory data for coffee and water for participant V1XS8. The analysis was conducted for the 7-s epochs after the inhale onset, prior to hand movements. ICA with the low-path filtering below 20 Hz (*U* = 1883, *p* < 0.008 for a peak around 10 Hz in “vanilla” and “water” conditions is significant at Bonferroni corrected level).

In several subjects, we observed clear EEG responses to odor perception onset, which was also inhalation onset by task design. Since these responses corresponded to both olfactory processing and the act of inhalation; we cannot claim that those were purely olfactory-related patterns. Yet, we found differences in the responses to different odors, which indicated that these EEG patterns had an olfactory-related component. Non-parametric pairwise statistical testing of EEG patterns exhibited during processing of different odors to odorless control stimuli revealed significant differences in the components’ spectral power for some pairs (*p* < 0.05). After Bonferroni correction was applied to adjust the significance level for multiple comparisons, the significance level was set at *p* < 0.008 (0.05/6 tests). With these requirements, in two subjects significant differences were observed for the comparison of some of the odors to the odorless control for participants. The response to water was different from the response to coffee in subject A4SHD (*p* = 0.0074) and to vanilla in subject V1XS8 (*p* = 0.0043). Marginal significance (i.e., without Bonferroni correction) was observed for the comparison of water to citrus in subject V1XS8 (*p* = 0.04) and to coffee in subject O9ZEA (*p* = 0.04). These results are illustrated in Figures 12, 13.

### 4.3. Olfactory related evoked responses during the final report

**FIGURE 14:**
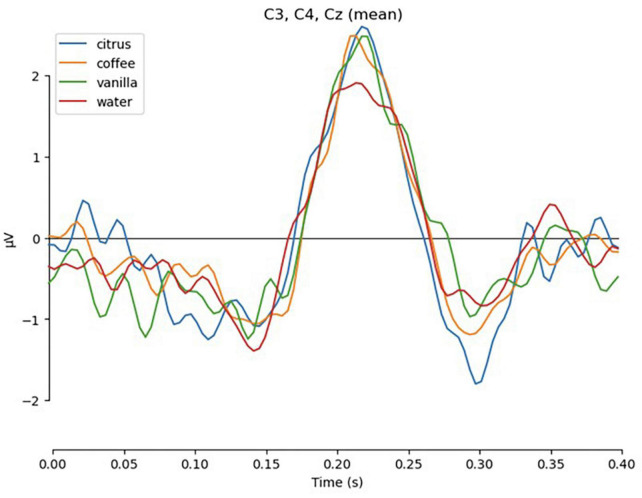
Evoked response to the presentation of final response targets, averaged across all subjects and channels C3, C4, Cz.

Clear evoked responses were found for the final appearance of the set of response targets. For this analysis, we examined a 400-ms epoch following the appearance of the visual labels with baseline 1 s before the onset. The subjects observed the labels and pointed to one of the labels with the joystick. Those epochs represented the visual ERP for the labels and did not include movement related. As evident from the resulting grand average plot combining data for all subjects from the central channels C3, Cz, and C4 (Figure 14), the P200 response was suppressed in cases when no odor was perceived (condition “water”). This goes in line with previously reported data (Leleu et al., 2015).

The pairwise *t*-test performed on the individual evoked responses and the mean amplitude around the peak of P200 demonstrated statistically significant differences between each of the odors and the control condition: *t* = 2.78, *p* < 0.01 for citrus; *t* = 2.149, *p* < 0.05 for coffee; *t* = 2.114, *p* < 0.05 for vanilla.

An additional analysis of the peak-to-peak amplitude between P200 and N200 components revealed significant difference between the “citrus” and “water” conditions: *t* = 2.679, *p* < 0.05, while the differences between “vanilla” and “water” were at the margin of statistical significance: *t* = 1.754, *p* = 0.086. The differences between “coffee” and “water” conditions were insignificant: *t* = 1.338, *p* = 0.18.

## 5. Discussion

The main motivation of this work was the development of a home theater-like environment where visual, auditory and olfactory stimuli could be integrated. While controlling visual and auditory components of the environment is relatively easy, the delivery of odorants is more challenging, particularly when a near-natural olfactory environment is sought for. In our implementation, odors were delivered from the room ceiling and remained in the room for several seconds until they were removed using the ventilation system.

The behavioral paradigm that we used was an instructed-delay task where olfactory stimuli were injected in the room in the beginning of a trial followed by a period of odor assessment and the final report made with a joystick. With this approach, we were able to achieve stable perception of odors in healthy subjects without adaptation and deterioration in discrimination performance. This was clear from the mean performance accuracy for all participants (93.8%). In principle, similar results could be achieved with olfactory of a different design, provided that the subjects performed the same task. Yet, the settings proposed here are more natural, comfortable and controlled compared to both manual delivery of odors (Saha et al., 2013; Aydemir, 2017; Abbasi et al., 2019; Zhang et al., 2021; Ezzatdoost et al., 2020) and using an olfactometer attached to the nostrils (Murphy et al., 2000; Rombaux et al., 2006; Invitto and Mazzatenta, 2019). While the field has been growing of olfactory displays that achieve the stable perception of odors in a naturalistic setup (Nakamoto et al., 2012; Wen et al., 2018, 2019), only a few EEG studies employed such displays. Amores et al. (2018) conducted a study with a wearable olfactory display (an olfactory necklace) and recorded EEG data with a low-cost EEG headband. Fujiwara et al. (2021) and Morozova et al. (2022) used Aromajoin olfactory display in their studies with professional EEG recording. Our study extends this work by developing a natural environment where an olfactory display can be coupled with visual stimuli as well as stimuli of different modalities. As such, it can be used for a wide range of experiments employing EEG recordings an different behavioral tasks.

Overall, the proposed methodology is suitable for running a variety of olfactory-related tasks where clear perception of odors by the subjects is needed. The capacity of subjects to discriminate odors for a long time (up to 1.5 h or 120 trials) could be particularly useful in future research. Apparantly, olfactory adaptation (Dalton, 2000) was not an issue in our study, presumably because of different odors presented on different trials and attentional demands to the active discrimination task. We conclude that, with proper settings of the task, human subjects are capable of perceiving and discriminating odors for a long period of time.

The ICA analysis of the EEG recordings revealed clear olfactory-related neural patterns in one third of the subjects. Thus, olfactory-related responses were detected over the C4 electrode; these responses peaked at the frequency around 12 Hz. We suggest that these responses represent a mixture of a motor component related to the act of respiration and an odor-related component. The presence of the odor-related component was evident from the analysis that showed that different odors evoked different EEG patterns. As such, this component should be further explored in order to develop a robust olfactory-based BCI system.

Curiously, even during the final stage of the task when motor reports were generated, the visual P200 EEG component produced in response to the presentation of the report targets was modulated depending on the odor being perceived. Specifically, the amplitude of the P200 response was lower for the no-odor condition. This is significant because the P200 component is known to be crucial for primary categorization and attention-related processes (Luck and Hillyard, 1994). Here, the subjects could have categorized the presence of an odor versus its absence into two different categories. Thus, this P200 could provide odor discrimination-related information for an olfactory-based BCI.

Overall, the incorporation of the odor delivery system into a home theater-like environment and running an instruction-delay odor-discrimination task while EEG and respiration are being recorded allowed us to analyze neural processing of odors in healthy humans. The experimental setup can be upgraded in the future to incorporate additional cross-modal interactions and more sophisticated cognitive tasks. Furthermore, the same setup can be used for constructing BCI paradigms, where olfactory-related decisions are decoded and transmitted to the computer, and neurofeedback systems, where odors are used as indicators of activity changes in various brain areas. Using such BCIs and neurofeedback systems is particularly appealing as rehabilitation tools for people suffering from neural disabilities.

## Data availability statement

The raw data supporting the conclusions of this article will be made available by the authors, without undue reservation.

## Ethics statement

The studies involving human participants were reviewed and approved by the Higher School of Economics Institutional Review Board. The patients/participants provided their written informed consent to participate in this study.

## Author contributions

IN, NB, and ML designed the study. IN and NB performed the experiments. IN and DK analyzed the data. IN, DK, and ML wrote the manuscript. All authors contributed to the article and approved the submitted version.
