# Supplementary material for: An experimental paradigm for studying EEG correlates of olfactory discrimination
Source: Front Hum Neurosci. 2023 May 25;17:1117801. doi: 10.3389/fnhum.2023.1117801 (PMC10248234; doi:10.3389/fnhum.2023.1117801)
Supplement: Supplementary file 1 [file Table_1.docx]

Supplementary Material

An experimental paradigm for studying EEG correlates of olfactory processing during an instructed-delay task

**Ivan Ninenko^1,2*^, Daria F. Kleeva^1^, Nikita Bukreev^3^, Mikhail A. Lebedev^1^**

^1^ Institute for Cognitive Neuroscience, HSE University, Moscow, Russia

^2^ V. Zelman Center for Neurobiology and Brain Rehabilitation, Skolkovo Institute of Science and Technology, Moscow, Russia

^3^ Sensorylab, Inc., Moscow, Russia

*** Correspondence:**Corresponding Author
ivan.ninenko@gmail.com

# Supplementary Tables

Table 1. Detection of inhalation onsets in different subjects.

| **Subject** | **# detected inhales (out of 120)** | **% detected inhales** |
| --- | --- | --- |
| A4SHD_21F | 120 | 100.0% |
| V1XS8_31M | 119 | 99.2% |
| 9V6NM_36M | 118 | 98.3% |
| ZU9B3_34M | 118 | 98.3% |
| ZNH6Y_22M | 118 | 98.3% |
| 5V005_22M | 114 | 95.0% |
| 6K0UR_33M | 111 | 92.5% |
| 24WAG_31M | 111 | 92.5% |
| FK7CK_40M | 102 | 85.0% |
| VRWV7_31F | 100 | 83.3% |
| 7PF8Z_36F | 89 | 74.2% |
| O9ZEA_23F | 88 | 73.3% |
| VFDSF_20M | 85 | 70.8% |
| 3KACQ_44M | 76 | 63.3% |
| 99CEM_22F | 58 | 48.3% |
| 1R912_39F | 39 | 32.5% |

Table 2. Performance accuracy across the participants.

| **Subject** | **# correct trials**  **(out of 80)** | **% of correct trials** |
| --- | --- | --- |
| ZU9B3 | 79 | 98.8% |
| V1XS8 | 79 | 98.8% |
| 9V6NM | 79 | 98.8% |
| ZNH6Y | 77 | 96.3% |
| O9ZEA | 77 | 96.3% |
| VRWV7 | 76 | 95.0% |
| 5V005 | 76 | 95.0% |
| A4SHD | 75 | 93.8% |
| 24WAG | 75 | 93.8% |
| 1R912 | 75 | 93.8% |
| 3G2BW | 74 | 92.5% |
| 7PF8Z | 73 | 91.3% |
| 99CEM | 70 | 87.5% |
| 3KACQ | 69 | 86.3% |
| VFDSF | 58 | 72.5% |
| 6K0UR | 53 | 66.3% |
| FK7CK | 35 | 43.8% |
